# Fair Harmonization. How can we harmonize digital cognitive tools while also preserving local culture and context?

**DOI:** 10.1002/alz70857_097258

**Published:** 2025-12-24

**Authors:** Karen Blackmon

**Affiliations:** ^1^ Brain and Mind Institute, Aga Khan University, Nairobi, Kenya; Geisel School of Medicine at Dartmouth, Hanover, NH, USA

## Abstract

An internationally harmonized and cross‐culturally relevant brain health index is needed for accurate global tracking and forecasting of brain health loss attributable to infectious diseases, high‐risk human behaviors, and environmental exposures. The index should be able to: (1) absorb advances in digital assessment tools; (2) quantify brain health at the level of the individual and population; (3) characterize the burden, severity, and profile of brain health loss; (4) track changes in brain health over time and as the result of interventions; and (5) preserve sociocultural norms and linguistic diversity. An International Brain Health Index (IMBHI) is proposed to accomplish these aims by leveraging the Alkire‐Foster counting method, which has successfully harmonized poverty estimation across financially, geographically, and culturally diverse settings. This approach allows for flexibility at the level of indicators and cut‐off values, while harmonizing domains, healthy control criterion, and an actuarial approach to classifying brain health loss. The stepwise process for deriving the IBHI includes Step 1. Select setting and unit of analysis (individual/population). Step 2. Select brain health domains. Step 3. Select brain health indicators to assess each domain (cognitive tests, scales, and digital tools validated in the target setting). Step 4. Utilize context‐specific indicator cut‐offs for a “low” score, defined by downward deviation from local normative reference groups. Step 5. Establish brain health domain deprivations. If more than one indicator is classified as “low” within a domain, then classify as “deprived”. Step 6. Sum domain deprivations, then divide by the total number of domains to arrive at a percentage of brain health deprivations. Step 7. Classify “brain health loss” by determining whether the percentage of brain health domain deprivations exceeds established thresholds. Step 8. Calculate the total burden, severity, and profile of brain health loss. The IBHI has the potential to model the individual‐ and population‐level burden of brain health loss across LMICs. By allowing for local autonomy in indicator selection, it overcomes barriers to absorption of novel digital tools and addresses measurement imprecision in regions where existing neuropsychological tools have limited validity. This approach to fair harmonization is essential for advancement towards global brain health equity.

